# Mediastinal hematoma following transradial percutaneous coronary intervention: case report and literature review

**DOI:** 10.3389/fcvm.2025.1414907

**Published:** 2025-03-07

**Authors:** Qiangsong Jin, Jiamin Wang, Xiaogang Hu, Xianqing Hu, Shenwen Fu

**Affiliations:** Department of Cardiology, Affiliated Jinhua Hospital, Zhejiang University School of Medicine, Jinhua, China

**Keywords:** percutaneous coronary intervention, radial artery, complication, mediastinal hematoma, interventional embolization

## Abstract

Mediastinal hematoma due to transradial PCI is rare. We reported a case of chest tightness, dyspnea, progressive neck swelling after transradial PCI. Clinical examinations such as chest computer tomography were completed and identified as mediastinal hematoma caused by a rupture of the subclavian artery branch and occlude the artery under digital subtraction angiography guidance, the artery was considered to be a collateral vessel of non-bronchial arterial circulation. This case highlights the necessity of exercising extreme caution when utilizing hydrophilic-coated curved-tip guidewires during the advancement process in peripheral vascular procedures. Mediastinal hematoma is a life-threatening complication and progresses rapidly, we need timely identification and diagnosis based on symptoms and appropriate clinical examination, interventional embolization therapy is critical for patients with poor response of conservative treatment.

## Introduction

Percutaneous coronary intervention (PCI), as an effective treatment of coronary heart disease, has been widely applied in the world. With the gradual maturation and development of technology, transradial approach has gradually replaced the previous femoral artery access, benefiting from its fewer complications, better patient experience and comfortness, and shorter hospital stays, even reducing the mortality of patients with acute coronary syndrome (1–5). Currently, over 90% of PCI procedures in China are performed through the radial artery route (6, 7). Although radial artery access has many advantages, complications associated with it have gradually emerged in clinical practice (8). Mediastinal hematoma, as one of vascular rupture complications, is extremely rare in clinical practice, but can lead to serious consequences. This article describes a case of mediastinal hematoma after transraidal PCI and a series of diagnosis and treatment measures, along with a systematic review of the literature.

## Case report

A 58 years old Chinese man with a history of type 2 diabetes mellitus for many years and a long-term history of smoking. In 2016, he underwent implantation of two stents in the left anterior descending artery (LAD) due to “acute anterior wall ST-segment elevation myocardial infarction”, followed by another stent implantation in the left circumflex artery (LCX) 1 month later. He has been regularly taking antiplatelet aggregation and lipid-lowering drugs after surgery. In November 2022, he experienced chest pain again, with profuse sweating, and presented to our hospital for consultation. He was diagnosed with acute non-ST-segment elevation myocardial infarction (NSTEMI). Coronary angiography (CAG) via the right radial artery showed that the original stents in the LAD and LCX were patent, the obtuse marginal branch (OM) was completely occluded, and the right coronary artery (RCA) had severe stenosis but was relatively short. One stent was implanted after opening the OM, and the patient received regular treatment with aspirin and clopidogrel dual antiplatelet therapy, along with other conventional medications. A month later, the patient still felt chest tightness and requested further treatment for the RCA lesion, leading to admission to the hospital.

**Figure 1:**
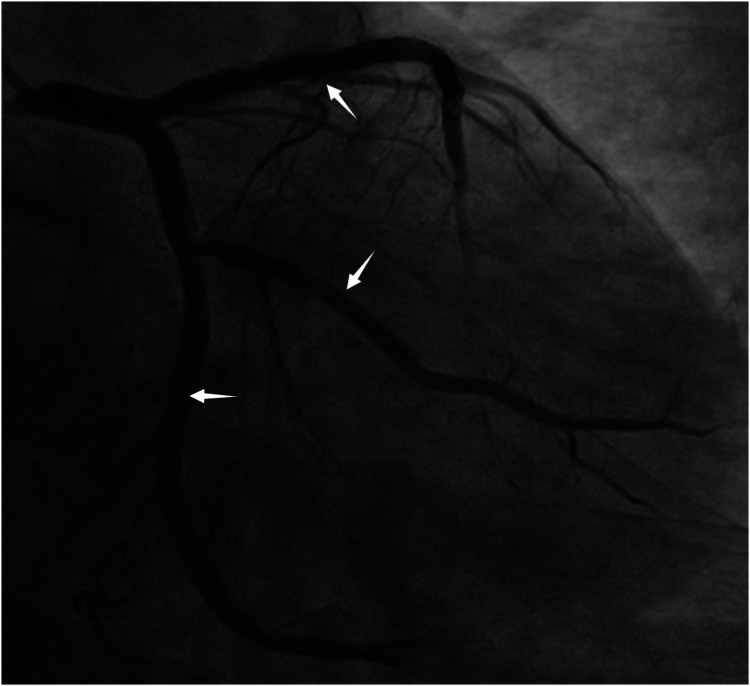
The original stents in the LAD, LCX, and OM are patent (red arrow indicates the LAD stents, white arrow indicates the OM stent, black arrow indicates the LCX stent).

**Figure 2:**
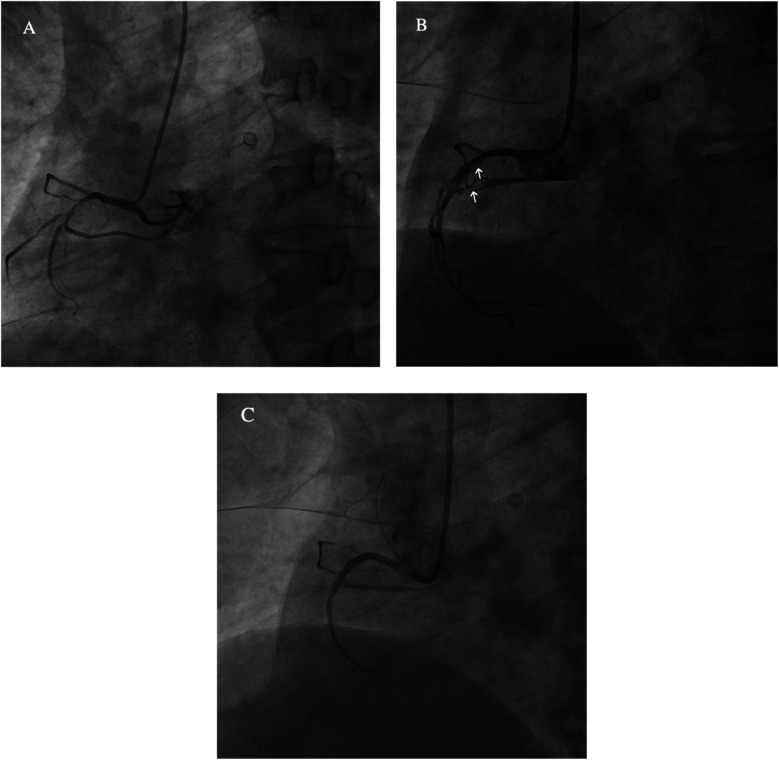
**(A)** The right coronary artery (RCA) is short and severely narrowed in the proximal segment. **(B)** After drug balloon dilation, coronary artery dissection is observed (white arrow). **(C)** Post-stent implantation, the stent is well-expanded, with TIMI III flow.

Preoperative assessment showed no significant abnormalities in routine biochemical indicators such as blood routine and coagulation function. Chest CT indicated bilateral pulmonary emphysema. After a comprehensive evaluation of the patient's vascular access, a 6F short sheath (Terumo, Tokyo, Japan) was inserted through the right radial artery. Under x-ray fluoroscopy guidance, a 5F JR4.0 catheter (Cordis, Chihuahua, Mexico) was advanced with the assistance of a Hydrophilic Guide Wire (length: 180 cm, angled tip, curvature tip length: 3 cm, tip diameter 0.035″, Merit, Utah, USA) for CAG (during which inadvertent entry of the wire tip into the right subclavian artery branch occurred twice, promptly retracted without discomfort from the patient). The angiography revealed patent stents in the LAD, LCX, and OM (Figure 1), and severe stenosis in the proximal RCA (Figure 2A). With the patient's consent, intervention for the RCA was performed. A 6F JR4.0 guiding catheter (Medtronic, Minneapolis, USA) was exchanged, and a Sion guidewire (Asahi, Aichi, Japan) was selected to reach the distal RCA. A Runthrough NS guidewire (Terumo, Tokyo, Japan) was advanced to protect the OM. A 2.0 × 15 mm Sprinter balloon (Medtronic, Minneapolis, USA) was inflated to 16 atm, followed by a 2.25 × 20 mm RESTORE DEB Paclitaxel balloon (Cardionovum, Am Bonner Bogen2, Germany) at 10 atm for 60 s. Subsequent angiography revealed dissections in the proximal RCA and OM (Figure 2B). A 2.25 × 30 mm Resolute integret stent (Medtronic, Minneapolis, USA) was implanted at 8 atm, followed by inflation with a 2.5 × 12 NC Sprinter balloon (Medtronic, Minneapolis, USA) to 12 atm within the stent. CAG confirmed no residual stenosis, with TIMI III flow (Figure 2C). The patient remained asymptomatic, and the procedure was concluded. The radial artery sheath was removed. The total procedure time was 77 min (CAG + PCI), and a total of 8,000 units of heparin were used.

**Figure 3:**
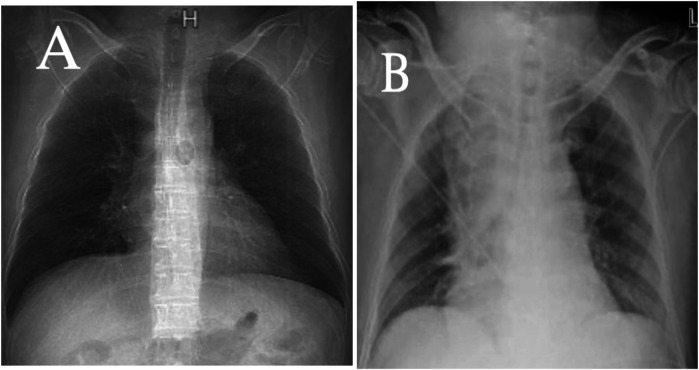
A comparison of chest x-rays taken the day before surgery **(A)** and on the day of surgery **(B)** shows a significant widening of the mediastinum in **(B)**.

Approximately 1 h post operation, the patient complained of progressively worsening chest pain located behind the sternum, accompanied by sweating, coughing with white sputum, wheezing in the throat, and neck swelling. His blood pressure was 144/89 mmHg, heart rate 63 bpm, and oxygen saturation 98%. Bedside electrocardiogram showed no dynamic changes in ST-T compared to preoperative findings. Emergency bedside ultrasound revealed significant soft tissue swelling in the neck, up to 23 mm thick, without evident fluid collection. Emergency consultation with an otolaryngologist revealed marked mucosal edema on the pharyngeal wall, suggesting acute pharyngeal edema. After discussion, the patient received dexamethasone 5 mg intravenous (IV) and methylprednisolone 80 mg IV, along with nebulization of budesonide. The patient's cough and sputum alleviated, wheezing disappeared, and swelling did not worsen. Further chest x-ray on the same day revealed mediastinal widening compared to preoperative images (Figure 3), which was initially overlooked due to inadequate experience. Biochemical tests showed normal hemoglobin and cardiac enzyme levels. Two hours later, repeat testing showed persistent normal results. Considering the stable condition, aspirin and clopidogrel were not discontinued, and the patient was kept under conservative observation.

**Figure 4:**
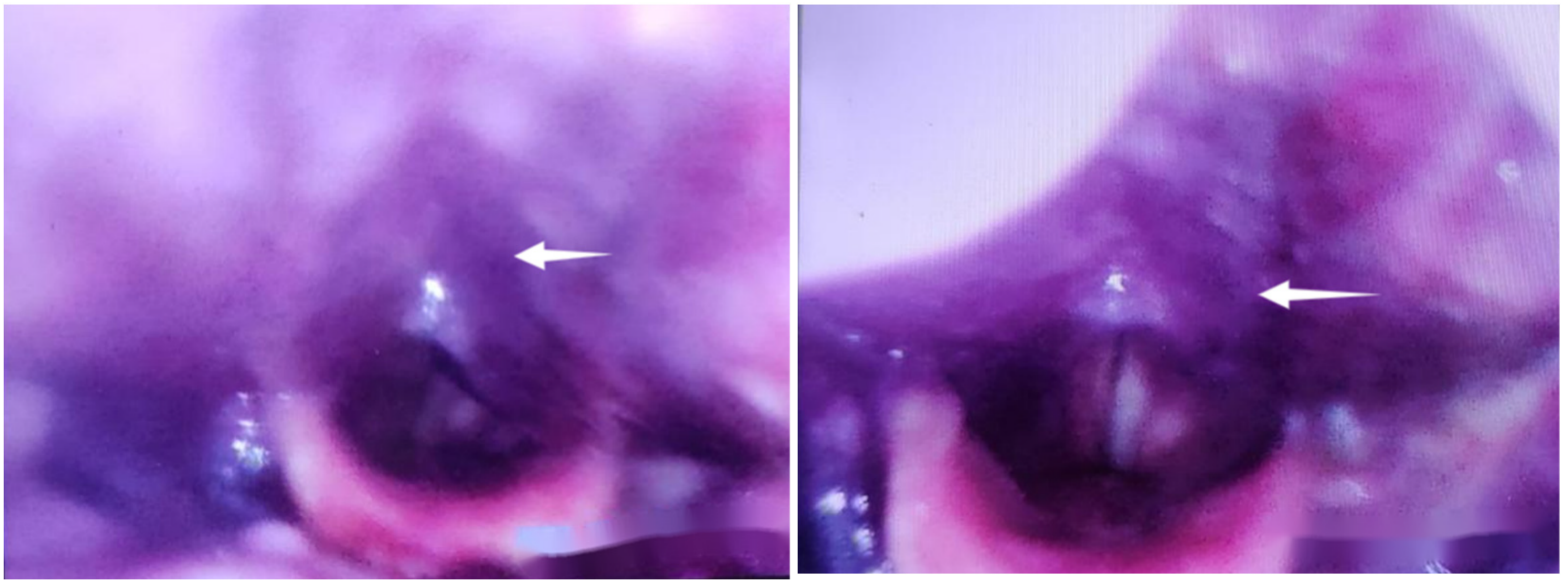
On the second day postoperatively, recurrence of symptoms, with electronic laryngoscopy revealing swelling of the pharyngeal wall accompanied by bluish discoloration.

**Figure 5:**
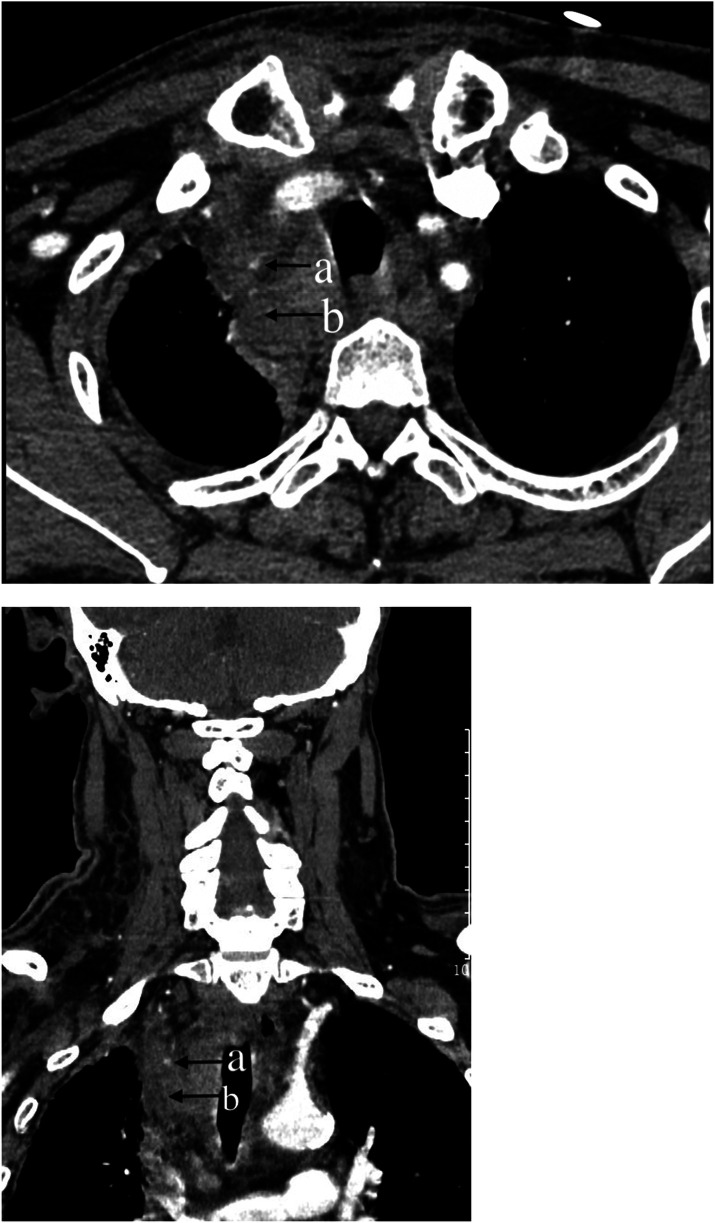
Chest computer tomography (CT) scan of the neck and chest: **(a)** Dot-like enhancement in the arterial phase, indicating active bleeding. **(b)** Mediastinal hematoma.

On the second post-PCI day, the patient experienced sudden chest tightness and shortness of breath again, with mouth breathing, but without exacerbation of neck swelling. Electrocardiographic monitoring showed sinus rhythm with a ventricular rate of 114 bpm, oxygen saturation of 92%, and blood pressure dropped to 90/57 mmHg. Oxygen therapy at 3l/min alleviated chest tightness, and 10 min later with intravenous fluids blood pressure returned to 121/71 mmHg. Repeat hemoglobin testing showed a decrease from 154 g/L to 123 g/L, with normal coagulation function. Otolaryngologic examination revealed persistent mucosal swelling and bluish discoloration, suggestive of hemorrhagic changes (Figure 4). Emergency Chest computer tomography (CT) scan showed swelling of the posterior pharyngeal wall and vocal cords, upper mediastinal hematoma, and signs of active bleeding, without evidence of aortic dissection (Figure 5). Considering the risk of further hematoma enlargement and airway obstruction, emergency digital subtraction angiography (DSA) was planned to identify the ruptured vessel after communication with the patient's family.

**Figure 6:**
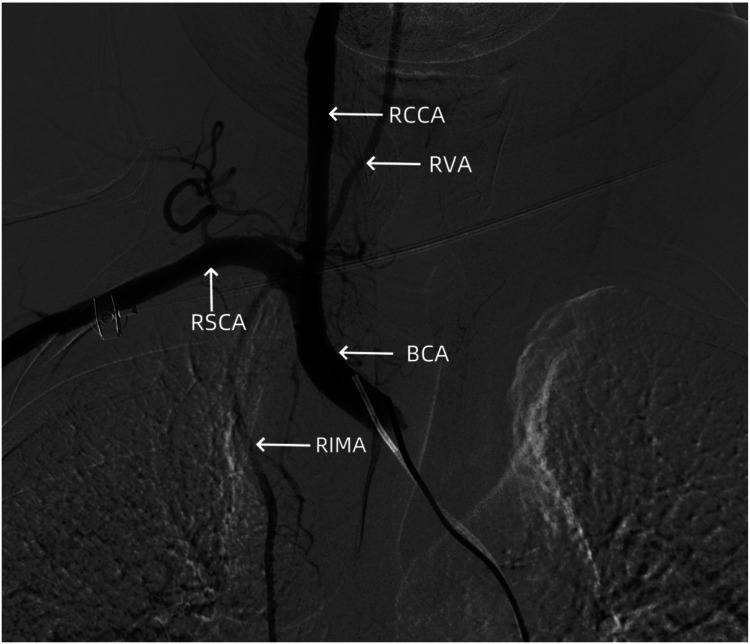
Angiography showing no signs of dissection hematoma or perforation bleeding in the brachiocephalic trunk, cervical internal carotid artery, and subclavian artery. RCCA, right common carotid artery; RVA, right vertebral artery; RSCA, right subclavian artery; BCA, brachiocephalic artery; RIMA, right internal mammary artery.

**Figure 7:**
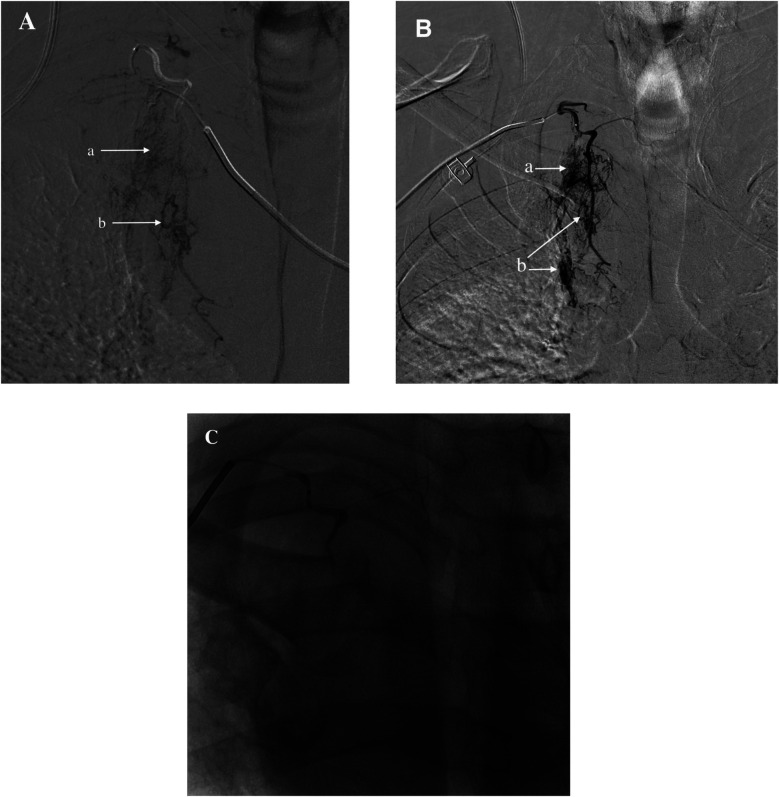
**(A)** Identified responsible artery via the femoral artery route, but catheter fixation was not possible. **(B)** Identified responsible artery via the right radial artery route. **(C)** Completion of embolization. **(a)** Diffuse extravasation of contrast agent indicating bleeding. **(b)** Shunt to systemic pulmonary artery.

The patient's previous clinical records indicated occlusion of the right iliac artery. Therefore, the left femoral artery route was chosen. With a 5 F sheath, a 5 F pigtail catheter (Cordis, Chihuahua, Mexico) was advanced to the aortic arch for angiography, which showed no signs of obvious bleeding or dissection (Figure 6). Subsequently, after exchanging for a Hunter1 catheter (Cordis, Chihuahua, Mexico), a Merit Maestro microcatheter (Merit, Utah, USA) was inserted into the right subclavian artery to explore the responsible branch artery. Although the responsible artery was identified successfully, the catheter could not be secured at the opening of the responsible artery (Figure 7A). Therefore, the righr radial artery was used, and after the microcatheter entered the branch vessel, angiography showed extravasation of contrast agent, with clear visualization of the peripheral pulmonary artery vascular network (Figures 7A,B). Polyvinyl alcohol (PVA) particle embolic agents (diameter: 350–560 μm, Alicon, Hangzhou, China) and gelatin sponge particle embolic agents (diameter: 560–710 μm, Alicon, Hangzhou, China) were slowly injected through the microcatheter until the blood flow in the responsible artery completely stopped. Subsequent angiography confirmed complete embolization (Figure 7C), and the procedure was concluded.

**Figure 8:**
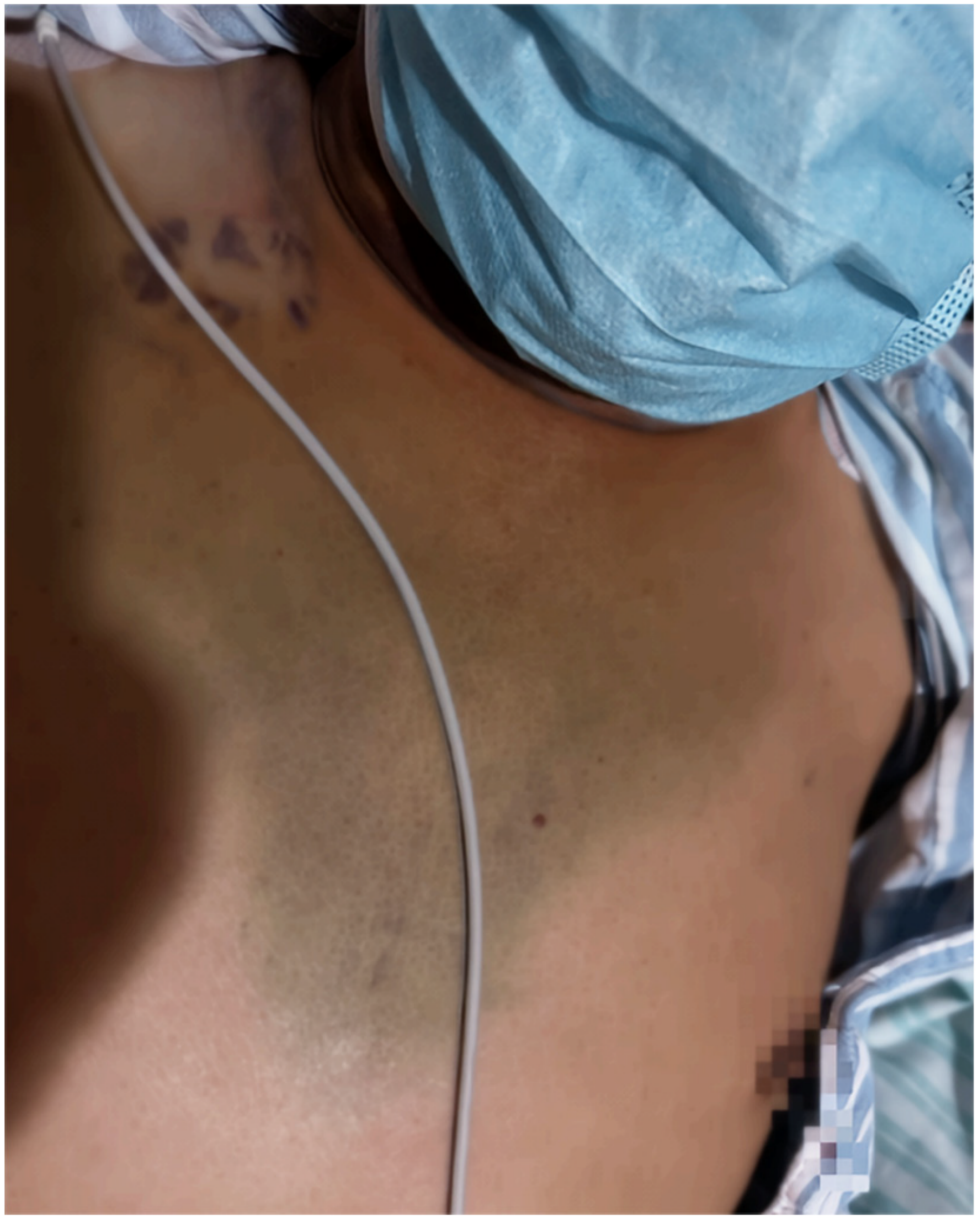
On the 6th day post PCI, bruising is visible in the chest anterior region.

**Figure 9:**
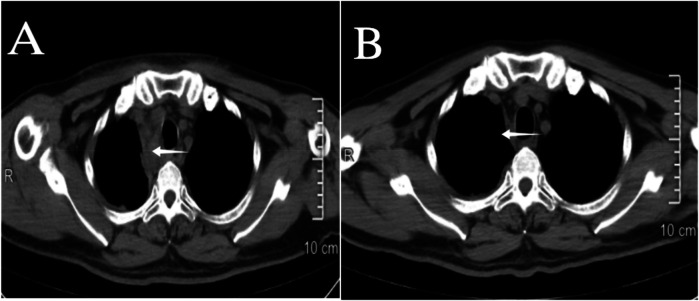
**(A)** 2nd day post interventional embolization; **(B)** 12th day post interventional embolization, on the 12th day the mediastinal hematoma (indicated by the arrow ←) is nearly completely absorbed, and airway compression has improved.

Following the procedure, aspirin was immediately discontinued, and the patient was treated with clopidogrel alone for antiplatelet aggregation. The patient's chest tightness symptoms improved, cough and sputum production did not worsen, but significant throat pain was noted during swallowing, and neck swelling gradually decreased. Bruising was observed in the chest area (Figure 8). Dynamic monitoring of blood routine and chest CT showed a decrease in hemoglobin levels to a minimum of 103 g/L. Chest CT indicated gradual absorption of the mediastinal hematoma with secondary bilateral minimal pleural effusion. C-reactive protein (CRP) levels peaked at 147 mg/L. Symptomatic treatments for cough, sputum, and infection were provided based on the pulmonary condition. On the 7th day post-embolization, aspirin was reintroduced in combination with clopidogrel for dual antiplatelet therapy. A follow-up chest CT on the 13th day post-procedure showed almost complete absorption of the hematoma (Figure 9). The patient was discharged on the 21st day post-procedure. At 2 months post-discharge, the patient remains healthy.

## Discussion

Coronary artery intervention via the radial artery approach has been widely recommended by numerous guidelines (9, 10). Vascular-related complications occur in only 0.4%, with most incidents happening at the puncture site or limited to the radial artery segment (11). Compared to the femoral artery pathway, this approach indeed reduces many vascular-related complications, primarily due to the decrease in complications at the puncture site (8, 12). The radial artery approach allows for prompt and effective external management of complications at the puncture site and radial artery segment. However, for intraluminal segments of arteries such as the subclavian artery and the brachiocephalic trunk, where effective external compression cannot be applied, the consequences of perforation and rupture are usually catastrophic. According to Luo et al., among 126,625 patients who underwent coronary angiography from 2006 to 2013, there were only 9 cases of mediastinal hematoma caused by radial artery access postoperatively, with an incidence rate of only 0.74% (13). To our knowledge, although there have been individual case reports of mediastinal hematomas after PCI in recent years, most have been managed conservatively, including fluid supplementation, adjustment of anticoagulant drugs, and maintaining airway patency. Our case has a relatively comprehensive clinical diagnosis and treatment process, and there are few reports of successful embolization of responsible arteries to treat mediastinal hematomas similar to ours. Therefore, the diagnosis and treatment of such PCI complications have significant reference value.

In this case, we used a 0.035-inch hydrophilic-coated guidewire with a curved tip. The hydrophilic coating allows it to be in a super slippery state inside the body, facilitating its passage through twisted peripheral vascular segments. However, this characteristic also makes it prone to inadvertent entry into small branch vessels. Cases of mediastinal hematoma reported by Luo et al. mostly involved the use of this type of guidewire, and other scholars have also reported vascular injuries caused by this type of guidewire (14–16). Therefore, we recommend routinely using J-shaped non-hydrophilic guidewires for radial artery access procedures to reduce the chance of entering small branch vessels. For patients where J-shaped guidewires cannot pass due to vascular tortuosity, hydrophilic guidewires of this type may be considered. However, during the procedure, the tip of the guidewire must be adequately exposed under x-ray fluoroscopy, and gentle, slow movements should be ensured, avoiding excessive flicking of the tip to reduce the chance of entering branch arteries inadvertently. If entering the same branch artery multiple times, selective peripheral arterial angiography may be performed to evaluate vascular tortuosity and assess whether perforation or other injuries have occurred. The roadmap-guided guidewire delivery through complex and tortuous artery remains a viable option. In this case, selective angiography of the responsible vessel showed signs of bleeding as well as a systemic circulation-pulmonary artery fistula. Considering the patient's pulmonary examination findings, we speculate that this vessel is a secondary collateral vessel arising from chronic pulmonary disease. To our knowledge, there have been no reports of similar vessels causing mediastinal hematoma due to rupture. Although this vessel originates from a subclavian artery branch located within the mediastinum, it continues downward through the pleura and communicates with intrapulmonary vessels. If patients develop spontaneous hemoptysis or other pulmonary diseases later on, besides investigating the usual bronchial arteries, attention should also be paid to the possibility of bleeding from such vessels (17, 18).

The patient developed symptoms approximately 1–2 h after coronary angiography. Similar symptoms related to mediastinal hematoma have been reported to occur within 2 h in other cases, with some patients experiencing symptoms immediately during the procedure (13, 15, 19). Given the rapid progression of symptoms, early recognition and detection are crucial. The symptoms observed in this patient included chest tightness, neck swelling, accompanied by difficulty breathing and wheezing. Additionally, there was decreased skin oxygen saturation, and immediate examination with electronic fiberoptic laryngoscopy revealed significant mucosal edema in the throat. Acute allergic laryngeal edema was suspected, and symptomatic treatment with steroids resulted in symptom improvement, narrowing down our diagnostic approach. We speculated that the acute inflammatory edema reaction was induced by the rapid perfusion of blood into the narrow space of the neck mucosa due to acute bleeding. Symptom relief with similar medication has been demonstrated in cases reported by Nathaniel and SeongIl, indicating the potential efficacy of steroid therapy for post-PCI neck swelling (20, 21). However, symptoms of mediastinal hematoma still lack specific clinical manifestations, requiring consideration of specific intraoperative conditions, such as whether the guidewire has entered branch vessels or if there was forceful manipulation. Additionally, prompt completion of tests such as electrocardiography, blood tests, myocardial enzyme spectrum, blood gas analysis, and cardiac ultrasound is essential for timely differentiation from postoperative acute myocardial infarction, aortic dissection, cardiac rupture, contrast agent allergy reaction, etc. Imaging examinations are the most important means of distinguishing and diagnosing mediastinal hematomas. In this case, postoperative chest x-rays already indicated significant mediastinal widening, but due to lack of experience, attention was only focused on the presence of pulmonary edema, leading to delayed diagnosis. Neck and chest CT scans are specific tools for diagnosing neck hematoma and mediastinal hematoma, respectively. CT value measurements of abnormal fluid can assess the likelihood of hematoma, but if conditions permit, direct CT with contrast medium of the neck and chest is recommended. In addition to distinguishing from aortic dissection, it can also determine the location and extent of the hematoma and identify the ruptured vessel.

Considering the large size and extremely complex distribution of branches from the subclavian artery to the brachiocephalic trunk, surgical exploration to find the responsible vessel is challenging. Therefore, early completion of peripheral vascular DSA examination for patients with active bleeding is a reliable method. Considering the possibility of secondary injury with the original access route and the surgical habits of peripheral vascular interventionists, we initially chose the femoral artery to find the responsible artery. Although non-selective aortic angiography were performed successfully, due to the long distance from the femoral artery to the brachiocephalic trunk and the upward twist angle from the brachiocephalic trunk to the right subclavian artery, the microcatheter could find the entrance of the responsible vessel selectively, but due to the mismatch in the direction of the catheter tip and the entrance of the responsible vessel, there was insufficient support for further occlusion operations. After multiple failed attempts, we switched to the original right radial route, successfully and quickly performed selective angiography, and occluded the responsible artery. Therefore, from this case, we believe that when using DSA to find the cause of hematoma, completing non-selective aortic angiography via routes such as the femoral artery to confirm the presence of aortic dissection, even rupture, and ruling out major vascular injuries, followed by progressive selective vascular angiography to clearly assess branch artery injuries, is a feasible approach. If there is deviation between the entrance of the responsible artery and the direction of the catheter, or if the responsible artery cannot be found, we still recommend selecting the original route, allowing coronary interventionists to rely on memory to further confirm the approximate path of the loach guidewire, accelerating the search for the responsible vessel, and providing reliable guidance and support for subsequent occlusion operations, thereby achieving rapid and effective treatment.

## Conclusion

In summary, mediastinal hematoma, as a rare complication of transradial PCI, progresses rapidly and can have serious consequences. Caution should be exercised when using super-slippery hydrophilic guidewires during the intervention process. For patients with postoperative neck swelling accompanied by chest tightness and wheezing, the possibility of mediastinal hematoma should be considered, and prompt and thorough examination and evaluation should be performed. CT scans of the chest and neck are crucial for assessing the hematoma. Timely peripheral vascular DSA examination and occlusion embolization of the responsible bleeding vessel are necessary.

## Data Availability

The original contributions presented in the study are included in the article/Supplementary Material, further inquiries can be directed to the corresponding author.
